# Supplementary material for: Silencing of the Ortholog of DEFECTIVE IN ANTHER DEHISCENCE 1 Gene in the Woody Perennial Jatropha curcas Alters Flower and Fruit Development
Source: Int J Mol Sci. 2020 Nov 24;21(23):8923. doi: 10.3390/ijms21238923 (PMC7727821; doi:10.3390/ijms21238923)
Supplement: Supplementary file 1 [file ijms-21-08923-s001.pdf]

Supplementary Files

Silencing of the Ortholog of *DEFECTIVE IN ANTHER DEHISCENCE 1* Gene in the Woody Perennial *Jatropha curcas* Alters Flower and Fruit Development

Chuan-Jia Xu[1,2,3,†], Mei-Li Zhao[1,†], Mao-Sheng Chen[1,2,*], Zeng-Fu Xu[1,2,*]

1 CAS Key Laboratory of Tropical Plant Resources and Sustainable Use, Xishuangbanna Tropical Botanical Garden, The Innovative Academy of Seed Design, Chinese Academy of Sciences, Menglun, Mengla, Yunnan 666303, China; xuchuanjia@xtbg.ac.cn (C.-J.X.); zhaomeili@ xtbg.ac.cn (M.-L.Z.)

2 Center of Economic Botany, Core Botanical Gardens, Chinese Academy of Sciences, Menglun, Mengla, Yunnan 666303, China

3 College of Life Sciences, University of Chinese Academy of Sciences, Beijing 100049, China

† These authors contributed equally to this work

* Correspondence: chenms@xtbg.org.cn (M.-S.C.); zfxu@xtbg.ac.cn (Z.-F.X.)

Table S1. GenBank accession numbers of DAD1 proteins from various species in the phylogenetic tree shown in Figure 1B

Species	Protein	GenBank accession number
Arabidopsis lyrata	AlDAD1	XP_002880139.1
Eutrema salsugineum	EsDAD1	XP_006397662.2
Arabidopsis thaliana	AtDAD1	OAP10523.1
Capsella rubella	CrDAD1	XP_006293380.1
Carica papaya	CpDAD1	XP_021903310.1:44-429
Oryza sativa	OsEG1	XP_015640022.2
Ricinus communis	RcDAD1	XP_002511581.1:5-443

Jatropha curcas	JcDAD1	XP_012083862.1
Hevea brasiliensis	HbDAD1	XP_021641037.1:1-431

Table S2. Primers used in this study

Name	Sequence	Usage
XC108-F	TACTCGAGTCCGTCAATCAGATGGAGATAC	Construction of
XC109-R	TAGGTACCAGTTGGTTACAGAAGCGAGTAG	*JcDAD1*-RNAi vector
XC110-F	TAGGATCCTCCGTCAATCAGATGGAGATAC	Construction of
XC111-R	ATATCGATAGTTGGTTACAGAAGCGAGTAG	*JcDAD1*-RNAi vector
XE715-F	ATCAAGGAATCAATAATTGGGAGG	qRT-PCR of *JcDAD1*
XE716-R	GGTGGCATAAGTAGGTGAAGAAGG	
